# Continuous rectus sheath block in a patient with a postoperative rectus sheath hematoma: a case report

**DOI:** 10.1186/s40981-019-0236-z

**Published:** 2019-03-02

**Authors:** Sho Kumita, Shunsuke Tachibana, Takahiro Ichimiya, Michiaki Yamakage

**Affiliations:** 1 Department of Anesthesiology, Sapporo Medical University School of Medicine, South 1 West 13, Chuo-Ku, Sapporo, Hokkaido 090-8666 Japan; 2 Department of Anesthesia, Asahikawa City Hospital, 1-1-65 Kinseicho, Asahikawa, Hokkaido 070-8610 Japan

**Keywords:** Postoperative rectus sheath hematoma, Continuous rectus sheath block, Regional anesthesia

## Abstract

**Background:**

Severe abdominal pain caused by a rectus sheath hematoma (RSH) can decrease a patient’s activities of daily living. A case of postoperative RSH for which a continuous rectus sheath block (RSB) was effective is reported.

**Case presentation:**

A 62-year-old woman who had no previous medical history underwent hysterectomy, total cystectomy, and ileal conduit surgery for bladder cancer under epidural and general anesthesia. She complained of severe abdominal pain 40 min after removal of the epidural catheter on postoperative day (POD) 4. Computed tomography showed an RSH on POD 12. For pain relief, an ultrasound-guided continuous RSB was performed on POD 17. After the block, the numerical rating scale (NRS) score during movement decreased immediately (from 10 to 2 or 3), and she had no further need for oral or intravenous analgesics. She was discharged from the hospital without any complications on POD 28.

**Conclusions:**

Continuous RSB can be an effective technique for pain relief of postoperative RSH.

## Background

A rectus sheath hematoma (RSH) is caused by damage to epigastric vessels and/or the rectus muscle. There are various causes of such damage, such as abdominal surgery, trauma, anticoagulant therapy, and hematological disease [1, 2]. Conservative management is selected in hemodynamically stable RSH cases; however, abdominal pain is usually described as severe, sharp, and persistent [1]. Severe abdominal pain could decrease the patient’s activities of daily living (ADL) until the RSH disappears. There have been few studies and case reports of the efficacy of oral and intravenous analgesics or alternative pain management in conservative therapy for RSH.

Rectus sheath block (RSB) is often used as an analgesic technique for abdominal surgery. In this report, a case of postoperative RSH for which ultrasound-guided continuous RSB was effective is described.

## Case presentation

A 62-year-old woman (height 163 cm, weight 59 kg, BMI 22 kg/m^2^) with no previous medical history or history of medications was scheduled to undergo hysterectomy, total cystectomy, and ileal conduit surgery for bladder cancer. The patient was managed by epidural anesthesia (Th12/L1) and general anesthesia. Just after massive intraoperative bleeding, 1500 mL of colloid fluid, 8 units of red blood cells (RBCs) and 6 units of fresh frozen plasma (FFP) were administered. The total amount of bleeding was 3640 mL. After the surgery, she woke up smoothly, and extubation was performed in the operating room. She was then admitted to the intensive care unit (ICU). During her stay in the ICU, 2 more units of RBCs were provided. ICU staff noted that pain control by epidural anesthesia was sufficient, but she complained of nausea once. She was discharged from the ICU on postoperative day 1 (POD 1), and urologists started to manage her on their own ward (Table 1). She did not receive antithrombotic therapy for prevention of venous thrombosis postoperatively.

**Table 1:** Changes in the results of blood examinations

	Preoperation	Postoperation	POD 1	POD 11
WBC (/μL)	7550	12,100	14,330	8820
Hb (g/dL)	10.9	7.6	11.1	9.9
Plt (/μL)	309,000	101,000	113,000	305,000
Na (mEq/L)	142	144	142	139
K (mEq/L)	4.0	3.9	3.9	3.3
Ca (mg/mL)	9.8	7.5	7.7	8.9
BUN (mg/dL)	15.8	9.8	11.4	12.1
Cre (mg/dL)	0.83	0.96	0.99	0.81
TP (g/dL)	7.2	3.0	3.8	5.9
Alb (g/dL)	4.0	2.0	2.3	–
CK (IU/L)	48	66	121	–
PT-INR	0.99	–	–	–
APTT (s)	34.4	–	–	–

*PT-INR* international normalized ratio of prothrombin time

**Fig. 1:**
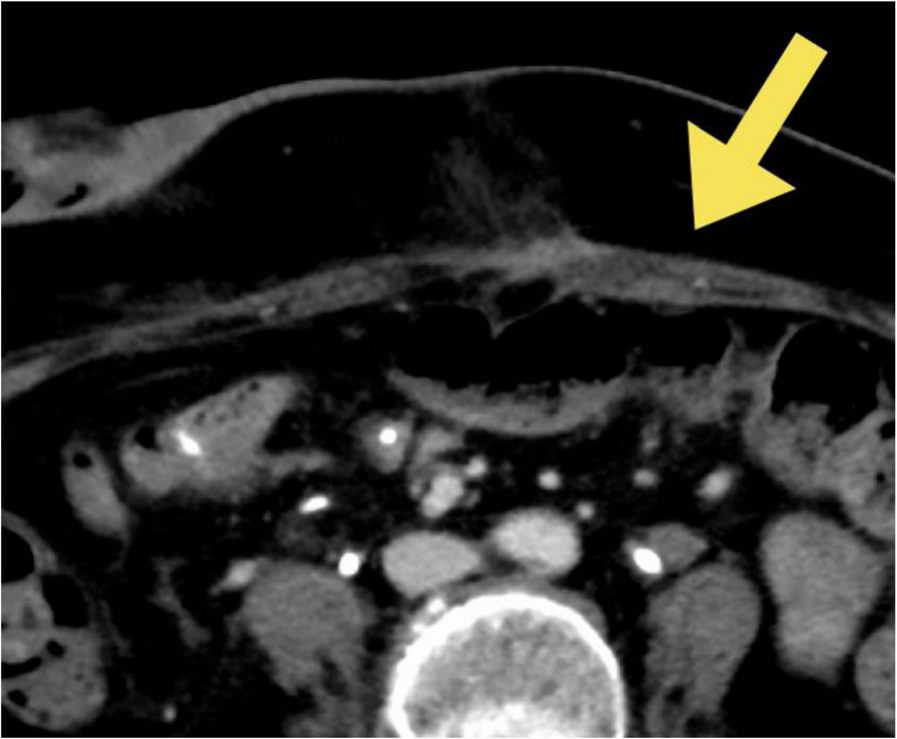
Enhanced abdominal CT on POD 16. The figure shows an enlarged left rectus abdominis with inhomogeneous contrast

On POD 4, she complained of severe abdominal pain (numerical rating scale (NRS) score: 10/10) 40 min after removal of the epidural catheter. She was unable to walk or eat despite pain relief provided by intravenous infusion of acetaminophen and NSAIDs. On POD 12, computed tomography (CT) showed a hematoma extending from inside the rectus muscle to subcutaneous tissue, and the surgeon started to administer tramadol (50 mg/day). On POD 16, CT showed that the size of the RSH was unchanged (Fig. 1), and the NRS score was high (resting: 2/10, moving: 10/10). The anesthesiologists were consulted regarding pain management. On POD 17, a rectus sheath catheter was placed under ultrasound guidance. A high-frequency linear probe was placed transversely with the rectus muscle immediately lateral to the umbilicus. An 18-G Tuohy needle was inserted in-plane to the US transducer and advanced until the tip had reached the posterior rectus sheath. Then, 20 mL of 0.375% ropivacaine were injected, and the catheter was passed through the needle to 5 cm within the rectus sheath. A total of 150 mL of analgesics (146 mL of 0.2% ropivacaine and 4 mL of fentanyl at 200 μg) were continuously administered at a rate of 4 mL/h. The NRS score decreased immediately just after ropivacaine injection (resting 0/10, moving 2 or 3/10), and she was able to walk and eat. She had no further need for oral or intravenous analgesics after the block procedure. After removing the catheter on POD 28, she had no complaints of abdominal pain and was discharged from the hospital without catheter site infection.

## Discussion

Pain in a case of RSH was successfully relieved by RSB. It was shown that continuous RSB can be an effective analgesic technique for postoperative RSH. According to a review of RSH [1], abdominal pain is usually reported as severe, sharp, and persistent. Conservative management can be selected in hemodynamically stable patients if the hematoma stops expanding. However, in the present case, the patient could hardly walk or eat for about 2 weeks. A previous study showed that ultrasound-guided continuous RSB provides effective postoperative analgesia equivalent to that of epidural anesthesia even in open abdominal surgery [3]. In addition, RSB has the potential benefit of avoiding the critical complications of epidural anesthesia, such as hematoma, nerve injury, motor weakness, and hypotension [4].

There were several possible causes of the RSH in the present case: surgical procedures, loss of coagulation factors due to intraoperative bleeding, and twisting of the rectus muscle caused by postoperative nausea and vomiting. In the surgical process, it was likely that massive bleeding caused a hemorrhagic tendency due to loss of coagulation factors. Moreover, the patient was administered 1500 mL of colloid fluid before FFP administration, which might have caused temporary dilutional coagulopathy. It is unknown why and when RSH occurred; however, even if the surgeons did not cause direct damage to the inferior epigastric artery, retractors can also damage the rectus muscle or branches of vessels and cause RSH in a patient with such a hemorrhagic tendency.

**Fig. 2:**
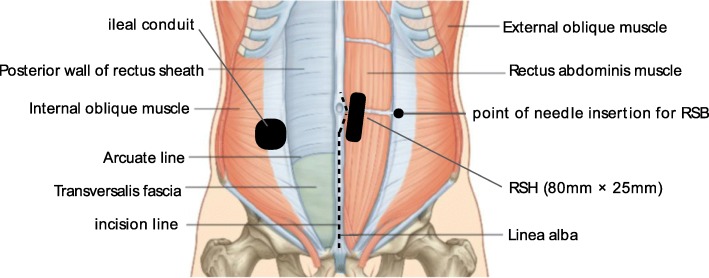
Schema of the perioperative abdominal wall and site of the incision line, ileal conduit, RSH, and RSB in this case. The figure shows the site of the incision line, ileal conduit, RSH, and point of needle insertion for RSB

For safe block procedures, ultrasound guidance enables administration of a local anesthetic and placement of a catheter into the correct plane, and it reduces the risk of peritoneal and vascular punctures. Below the arcuate line, the posterior rectus sheath is absent, and RSH therefore tends to extend beyond the midline and posteriorly [1]. Moreover, it increases the risk of peritoneal puncture. In the present case, RSH developed immediately left lateral to the umbilicus (Fig. 2). When RSB was performed, the site of RSH was checked, and the needle could be safely inserted into the lateral umbilicus with ultrasound guidance. A hematoma is generally one of the risk factors for surgical site infection [5]. Therefore, care must be taken regarding the location of the catheter and infectious spread around the catheter. Local anesthetic agents have vasodilating actions [6]. Therefore, ropivacaine may release the spasm of the inferior epigastric artery to cause re-bleeding. In this case, whether the inferior epigastric artery or branches had been damaged in the perioperative period could not be checked. However, we should determine the vital signs and size of the RSH after continuous RSB to detect re-bleeding. In addition, RSB should not be performed together with anticoagulant therapy.

For RSH patients, we should consider RSB if there is no active bleeding or hemorrhagic tendency. Severe abdominal pain can continue for several weeks, and it can greatly degrade patients’ ADL. Some postoperative RSH cases may remain unrecognized, and there may be many “hidden” postoperative RSH cases. Further studies on the efficacy and safety of continuous RSB for RSH cases are needed.

In conclusion, continuous RSB can be an effective analgesic technique for postoperative RSH.

## Abbreviations

ADL: Activities of daily living
CT: Computed tomography
FFP: Fresh frozen plasma
ICU: Intensive care unit
NRS: Numerical rating scale
POD: Postoperative day
RBCs: Red blood cells
RSB: Rectus sheath block
RSH: Rectus sheath hematoma
